# Photoconversion of Purified Fluorescent Proteins and Dual-probe Optical Highlighting in Live Cells

**DOI:** 10.3791/1995

**Published:** 2010-06-26

**Authors:** Gert-Jan Kremers, David Piston

**Affiliations:** Department of Molecular Physiology and Biophysics, Vanderbilt University

## Abstract

Photoconvertible fluorescent proteins (pc-FPs) are a class of fluorescent proteins with "optical highlighter" capability, meaning that the color of fluorescence can be changed by exposure to light of a specific wavelength. Optical highlighting allows noninvasive marking of a subpopulation of fluorescent molecules, and is therefore ideal for tracking single cells or organelles.

Critical parameters for efficient photoconversion are the intensity and the exposure time of the photoconversion light. If the intensity is too low, photoconversion will be slow or not occur at all. On the other hand, too much intensity or too long exposure can photobleach the protein and thereby reduce the efficiency of photoconversion.

This protocol describes a general approach how to set up a confocal laser scanning microscope for pc-FP photoconversion applications. First, we describe a procedure for preparing purified protein droplet samples. This sample format is very convenient for studying the photophysical behavior of fluorescent proteins under the microscope. Second, we will use the protein droplet sample to show how to configure the microscope for photoconversion. And finally, we will show how to perform optical highlighting in live cells, including dual-probe optical highlighting with mOrange2 and Dronpa.

**Figure:**
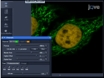


## Protocol

### 1. Preparation of fluorescent protein droplet samples

A fluorescent protein droplet sample consists of a 1-octanol/water emulsion with the fluorescent protein residing in the water phase. This emulsion is sandwiched between a microscope slide and a 22 mm square cover glass for microscopy applications.

1. Before making fluorescent protein droplet samples the microscope slides and cover glasses need to be cleaned and coated with a hydrophobic agent.
2. Clean glassware by washing 5 minutes with acetone and leave to dry by air. (Optionally, after cleaning the glassware can be treated for 30 seconds in a plasma cleaner to obtain optimal coating results).
3. Prepare a 2% methyltrimethoxysilane solution in acetone and coat the glassware during a 2 minutes incubation in this solution. After coating remove the glassware from the solution and leave to dry by air. Then rinse with 70% ethanol from a spray bottle and leave to dry again. (Optionally, at this point the glassware can be baked for 1 hour at 80°C to covalently link the coating to the glassware). Coated glassware can be stored for at least one month.
4. Fluorescent proteins are purified as His_6_-tagged protein from *E. Coli*^1^. Measure the absorbance spectrum of the purified protein and prepare a stock dilution with an optical density of ~0.1 in STE buffer (150 mM NaCl, 10 mM Tris-HCl pH 8, 1 mM EDTA), containing 0.1% bovine serum albumin (BSA). In addition prepare 10 ml of a 1:1 mixture of 1-octanol and STE buffer in a 15 ml conical tube and mix vigorously. After mixing leave until the phase separation is complete. The top phase is the 1-octanol. (Caution: Because 1-octanol has a strong smell it is important to use a closed waste container for everything that comes in contact with 1-octanol.)
5. To make the emulsion pipette 45 μl 1-octanol and 5 μl fluorescent protein in an microfuge tube. Tap the tube a few times with your finger to start formation of the emulsion and then sonicate the tube for 30 seconds in a sonication bath. In the meanwhile get a coated microscope slide and cover glass ready. After sonication the emulsion should be completely cloudy. Immediately after sonication pipette 4 μl emulsion from the middle of the tube onto a coated microscope slide and cover with a coated cover glass.
6. If the procedure is done correctly the emulsion should spread evenly between the microscope slide and the object glass. Within minutes the sample should be stable, consisting of ~10 μm thick fluorescent droplets with varying diameter. The largest droplets are close to the center of the sample and the smaller are located further towards the edges.

### 2. Setting up a photoconversion experiment

The following procedure is a general strategy for setting up a fluorescent protein photoconversion experiment. This procedure can be applied for purified proteins as well as for live cells.

1. The following parameters provide a general starting point to set up your photoconversion experiment: 40x 1.3NA oil immersion objective Image size = 512 x 512 pixels Scan zoom = 4 Pixel dwell time = 6 μsec. Z-resolution (pinhole size) = 3 μm
2. Configure two detection channels for the initial and photoconverted fluorescence, as well as a "photoconversion channel". In this example we will use purified mOrange2 protein, which is a orange-to-red photoconvertible fluorescent protein. The orange species is detected using 561 nm excitation and the fluorescence is collected between 570 nm and 630 nm. The photoconverted red species is detected using 633 nm excitation and the fluorescence is collected between 640 nm and 700 nm. For the "photoconversion channel" select 488 nm excitation and collect fluorescence between 490 nm and 540 nm. (Note: imaging the photoconversion channel is not strictly necessary.)
3. Use the channel for imaging the initial fluorescence with continuous scanning to adjust the laser power and detector gain for optimal image quality.
4. Activate the photoconversion channel and select a low laser power. Start imaging a time lapse series and gradually increase the photoconversion laser until significant bleaching of the initial fluorescence is observed. Continue scanning until the initial fluorescence is approximately 75% bleached.
5. Deactivate the photoconversion channel and activate the detection channel for the photoconverted fluorescence. Start imaging with a high detector gain and low laser power and gradually increase the laser power until the photoconverted fluorescence is detected. Once you detect the photoconverted fluorescence you can adjust laser power and detector gain for optimal image quality.
6. Finally, the laser power used for photoconversion as well as the duration of photoconversion need to be optimized. Increasing the photoconversion laser power will accelerate the rate of photoconversion, however too much laser power will photobleach the protein.
7. Once the optimal photoconversion laser power and duration have been determined, these parameters can be used to configure a standard photobleaching or FRAP module and the "photoconversion channel" is no longer required.

### 3. Dual-probe optical highlighting with mOrange2 and Dronpa

Because of the red-shifted spectral properties, mOrange2 can be used in combination with the green photoswitchable fluorescent protein Dronpa for dual-probe optical highlighting to allow selective highlighting of 4 individual cell(organelle) populations.

1. Cells are grown in glass bottom MatTek dishes and transfected 24 hours prior to imaging using standard Lipofectamine2000 transfection^1^.
2. Set up the microscope for mOrange2 photoconversion as described in section 2.
3. Configure the microscope for Dronpa photoswitching. Dronpa fluorescence can be imaged using the mOrange2 "photoconversion channel" (see step 2.2). (Note: Minimize the laser power used for imaging Dronpa, because too much laser power will cause inactivation of Dronpa.) Add a channel for Dronpa photoactivation. We use 800 nm two-photon excitation for photoactivation, but alternatively this can be achieved using 405 nm excitation. Determine the laser power required for imaging, photoactivation, and photoinactivation of Dronpa fluorescence.
4. Caution: Photoconversion of mOrange2 and inactivation of Dronpa both occur upon 488 nm excitation. Because of the high laser power required for mOrange2 photoconversion this will also inactivate Dronpa fluorescence. On the other hand, Dronpa inactivation occurs already at much lower laser power and can be performed without significant mOrange2 photoconversion.
5. Once the parameters for mOrange2 photoconversion and Dronpa photoswitching are set, dual probe optical highlighting is achieved through the following steps. First, inactivate Dronpa fluorescence in the whole field of view with low power 488 nm excitation. Second, select a region of interest and photoconvert mOrange2 with high power 488 nm excitation. Finally, select a region of interest to activate Dronpa fluorescence.

### 4. Representative Results


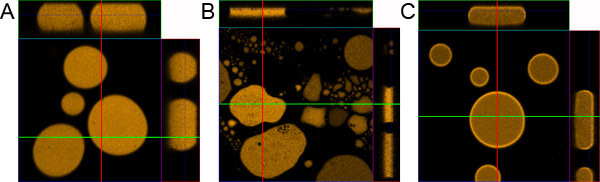
 **Figure 1. Droplet sample preparation.** A) Correctly prepared droplet sample. B) Sample prepared without coating the microscope slide and cover glass. C) Sample prepared without adding 0.1% BSA.


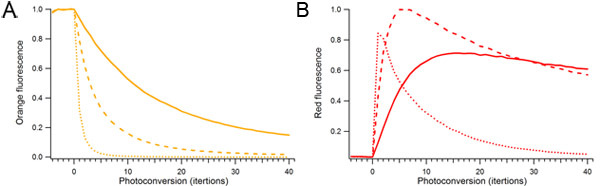
 **Figure 2. Effect of photoconversion laser power and duration on mOrange2 photoconversion.** Single droplets containing mOrange2 protein were continuously photoconverted using different amounts of 488 nm laser power. Relative laser power used for photoconversion was 10% (solid), 25% (dashed), and 100% (dotted). A) Orange fluorescent species. B) Photoconverted red fluorescent species.


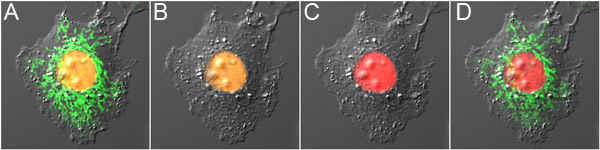
 **Figure 3. Dual-probe optical highlighting with mOrange2 and Dronpa.** A) Cell expressing mOrange2-Histone H2B and  Dronpa-Mito before photoconversion, showing orange fluorescence in the nucleus and green fluorescence in the mitochondria. B) Dronpa fluorescence was switched off with low power 488 nm excitation, causing minimal photoconversion of mOrange2. C) mOrange2 was photoconverted to red with high power 488 nm excitation.  D) Dronpa fluorescence was switched on again using 800 nm 2-photon excitation. The panels are overlays of the fluorescence images together with the differential interference contrast image. 

## Disclosures

No conflicts of interest declared.

## Discussion

The purified fluorescent protein droplet sample is a very convenient sample format for the photophysical characterization of fluorescent proteins, for example to study photobleaching kinetics and photoconversion kinetics.  The extremely small droplet volume (~20 picoliter) facilitates photobleaching and photoconversion experiments, which can be difficult to perform in cuvette based systems.  In addition, as shown here the droplet sample is ideally suited for setting up a confocal microscope for photoconversion applications.  The hydrophobic coating and the presence of BSA are important for obtaining homogeneous droplets. Without coating the droplets tend to be squashed against one of the glass surfaces and in the absence of BSA the fluorescent protein tend to accumulate at the 1-octanol/water interface, creating a halo of fluorescence (Figure 1). 

Fluorescent protein photoconversion is often regarded as an alternative to fluorescence recovery after photobleaching (FRAP), with the advantage that one can also follow the photoconverted species.  However, it is important to consider that photoconversion is critically dependent on the laser power used.  Too much photoconversion laser power or too long exposure will cause photobleaching rather than photoconversion, thereby reducing the amount of photoconverted fluorescence (Figure 2).

The red-shifted spectral properties of mOrange2 and its photoconverted species permit dual-probe optical highlighting with a green fluorescent optical highlighter. This can either be a photoswitchable fluorescent protein (Dronpa), as demonstrated here, or alternatively a photoactivatable fluorescent protein, for example PA-GFP.  Dronpa has the advantage that one can check its presence at the start of the experiment.  On the other hand, the use of Dronpa complicates optical highlighting, because all Dronpa fluorescence has to be inactivated first, and the fact that Dronpa fluorescence is gradually switched off during imaging.  These complications are less profound when using PA-GFP, but checking for the presence of PA-GFP before photoactivation can be more difficult.
